# Modification of pH Conferring Virucidal Activity on Dental Alginates

**DOI:** 10.3390/ma8041966

**Published:** 2015-04-21

**Authors:** Navina Nallamuthu, Michael Braden, John Oxford, David Williams, Mangala Patel

**Affiliations:** 1 Department of Oral Growth and Development, Queen Mary, University of London, London E1 4NS, UK; E-Mails: navina.ann@gmail.com (N.N.); michaelprf@aol.com (M.B.); 2 Queen Mary BioEnterprises, Innovation Centre, London E1 2AX, UK; E-Mail: j.s.oxford@retroscreen.com; 3 Centre for Clinical and Diagnostic Oral Sciences, Institute of Dentistry, London E1 2AT, UK; E-Mail: d.m.williams@qmul.ac.uk

**Keywords:** alginate, pH, antiviral, Herpes Simplex Virus type 1, magnesium oxide

## Abstract

To formulate an alginate dental impression material with virucidal properties, experimental alginate dental impression materials were developed and the formulations adjusted in order to study the effect on pH profiles during setting. Commercially available materials served as a comparison. Eight experimental materials were tested for antiviral activity against Herpes Simplex Virus type 1 (HSV-1). Changing the amount of magnesium oxide (MgO) used in the experimental formulations had a marked effect on pH. Increasing MgO concentration corresponded with increased pH values. All experimental materials brought about viral log reductions ranging between 0.5 and 4.0 over a period of 4 h. The material with the lowest pH was the most effective. The current work highlights the very important role of MgO in controlling pH profiles. This knowledge has been applied to the formulation of experimental alginates; where materials with pH values of approximately 4.2–4.4 are able to achieve a significant log reduction when assayed against HSV-1.

## 1. Introduction

Handling contaminated dental impressions presents an occupational health hazard to dental personnel, as it is a route of transmission of infectious micro-organisms from patients [1,2]. The rationale for disinfecting impressions is reinforced by reports on the persistence of viruses, bacteria and fungi, as discussed below.

Blood contamination of impressions presents a problem [3] and disinfection protocols effective against Hepatitis B and C, Herpes and HIV have been recommended [4,5]. Also of importance are the respiratory viruses Influenza A and B, Rhinovirus (common cold), Respiratory Syncytial Virus, Norovirus (causing diarrhoea and vomiting) and the blood-borne viruses Hepatitis C and B [6].

A wide range of persistent oral and environmental microorganisms also contaminate impressions and pose a serious health threat. Examples include Methicillin-resistant *Staphylococcus aureus* (MRSA), *Candida albicans* and *Pseudomonas aeruginosa* on the surface of alginate impressions, as well as the presence of viable micro-organisms on stone casts made with contaminated impressions [7,8].

The British Dental Association (BDA) states that dentists should assume responsibility for the cleaning and disinfection of impressions before dispatch to the laboratory, and have stipulated the following:

(1) Impressions must be rinsed under running water to remove saliva and debris. Heavily contaminated impressions should be cleaned using an ultrasonic bath with detergent.
(2) After cleaning, impressions should be disinfected following the manufacturer’s specification. Spray detergents are not recommended as they create an inhalation risk and are less effective than immersion disinfectants [9].

Although the BDA guidelines state that disinfection must be carried out, a specific protocol has not been recommended, thus disinfection techniques vary between dental practices. The differences in opinion on decontamination and disinfection procedures stem from the fact that conventional techniques can compromise both dimensional accuracy and surface quality of alginate impressions. This has been reported following immersion disinfection with commonly-used disinfectants including chlorhexidine [10], iodophor [11], phenol, formalin [12] and sodium hypochlorite, which can cause partial surface disintegration [13,14].

Alginates are hydrophilic materials and changes in osmotic potential following immersion in liquids mean they readily imbibe water, causing them to swell. The osmotic potential is reversed when water-soluble salts present in the alginate matrix are eluted, causing water to diffuse out and subsequent shrinkage of the material. Since these processes take place simultaneously, alginates are especially susceptible to unpredictable distortion following immersion disinfection [15]. This can compromise the accuracy of impressions, with a consequential adverse effect on clinical outcomes.

Much of the work carried out to date on disinfecting alginates has focussed on bacterial contaminants, but as previously mentioned, viruses also constitute a potential hazard to dental personnel. To test the proposition that it is possible to develop alginate impression materials with virucidal activity, we have selected HSV-1 as the target organism. HSV-1 is well-documented as causing an occupational health risk in dentistry [14,16,17]. The adopted strategy is to produce test materials ranging in pH, as the viral load can be substantially reduced by low pH values, possibly due to pH-dependent conformational changes in the virus that are induced outside the normal physiological pH range of 7.2–7.4 [18]. It is anticipated that decreasing the pH of alginate impression materials will confer virucidal activity, effectively creating self-disinfecting materials. This would be advantageous given the adverse effects observed with current disinfecting procedures.

Typically, alginate formulations include a sodium/potassium silicofluoride compound as a pH modifier [19,20] to ensure good reproducibility of the impression at the interface with the casting material. MgO has previously been identified as a cross-linking agent in dental alginates [21] and there is evidence that it also plays a significant role in the chemical reactions that govern the pH during the setting process. In this study, experimental alginates have been prepared and MgO content varied in order to investigate the effect on pH profiles during setting, and virucidal activity against HSV-1.

## 2. Results and Discussion

### 2.1. pH Measurements

**Figure 1:**
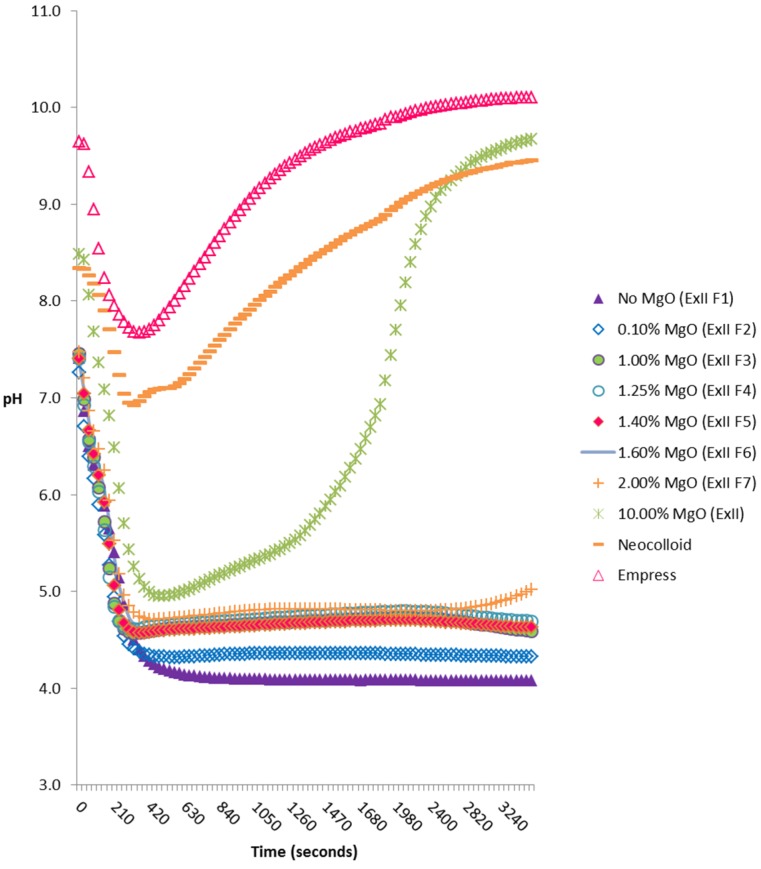
pH changes of Neocolloid, Empress and experimental alginates with varying MgO concentrations (0%–10%).

Figure 1 shows the variation of pH with time from mixing, for commercial and experimental materials. Error bars have been omitted for clarity and the maximum standard deviation between samples of the same material was 0.18. There were marked differences both in the degree of change in a given material and in the final pH values after 60 min. Typically, the pH of all materials decreased within five minutes after setting. The pH profiles of the commercial materials were similar, with a decrease for the first 5 min followed by a continuous increase, and both materials remained in the range of pH 7.0–10.0 for the duration of the experiment.

The pH profile for experimental material ExII was also similar to that of the commercial materials, except that a greater decrease in pH was observed (4.6 after 5 min). A continuous decrease to pH 4.1 was observed with ExII F1 for the first 10 min, after which the pH remained constant for the remainder of the experiment.

All other experimental materials (ExII F2–F7) decreased in pH in the first five minutes from ~8.5 (material containing 10% MgO) to ~4.2 (material containing no MgO). All materials with the exception of ExII (containing 10% MgO) had similar pH profiles and it was observed that increasing MgO concentration gave rise to a higher pH. It should be noted that the 10% MgO formulation had the highest pH and that containing no MgO had the lowest pH (~4.0).

Average pH values for all materials (at 60 min) were compared and denoted ‘s’ if statistically significant (*p* < 0.05), or ‘ns’ if not statistically significant (*p* > 0.05). The most apparent differences were seen with materials ExII (10% MgO), ExII F1 (no MgO) and ExII F2 (0.1% MgO). The differences in pH observed between these materials and all other materials, was statistically significant. The differences observed between materials ExII F3, ExII F4 and ExII F5 (1.0%, 1.25% and 1.4% MgO respectively) were not significant.

### 2.2. Virology

**Figure 2:**
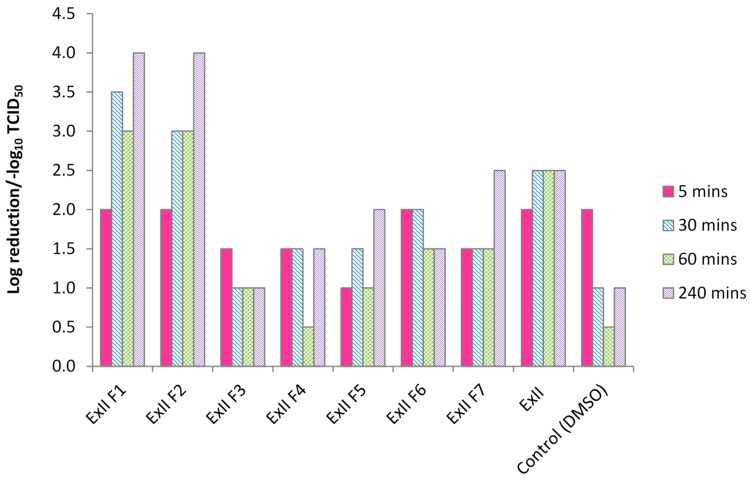
Log reduction/−log_10_TCID50 of HSV-1 with experimental alginate materials with varying MgO concentrations (0%–10%).

Log reduction/−log_10_TCID_50_ of HSV-1 achieved by experimental materials and the control (DMSO) at 5, 30, 60 and 240 min is shown in Figure 2.

For all materials (with the exception of ExII F3 and F6), virucidal activity increased with time. The greatest log reductions (4.0 (−log_10_TCID_50_)) were observed with materials ExII F1 and F2; the most acidic of all materials tested. Following this, ExII F7 and ExII (the most basic) both achieved log reductions of 2.5 (−log_10_TCID_50_). Materials ExII F3 and F4 were the least effective. A log reduction of 2.0 (−log_10_TCID_50_) was observed with the control.

Experimental alginates were formulated and their pH profiles compared to two commercially available materials. The formulation for the experimental material with the lowest pH profile (Ex11 F1) was then used to develop a further set of materials to take forward for antiviral testing against HSV-1.

### 2.3. pH Study

The pH changes shown in Figure 1 reflect the general trend of changes shown by Buchan and Peggie [19], namely a decreasing pH during setting, but with a wide range of actual pH values.

Of particular interest are the differences between ExII and ExII F1, where the omission of MgO has produced statistically significant decreases in pH. It is interesting to note that where various alginate formulations were studied previously [19], no mention of MgO was made. Thus, the use of MgO in alginate formulations may be a more recent development.

As previously demonstrated [21], MgO and potassium fluorotitanate (K_2_TiF_6_) are both hydrolysed, forming magnesium hydroxide (Mg(OH)_2_) and hydrofluoric acid (HF) respectively:

MgO + H_2_O → Mg(OH)_2_ (1)

K_2_TiF_6_ +2H_2_O → 2KF + TiO_2_ + 4HF (2)

The decrease in pH can be attributed to the formation of HF and the subsequent increase (seen in commercial materials and experimental formulations containing MgO) can be explained by the reaction of the two, where magnesium fluoride (MgF_2_) is formed:

Mg(OH)_2_ + 2HF = MgF_2_ + 2H_2_O (3)

Thus, in formulations where MgO is omitted, there is no Mg(OH)_2_ produced, so the HF is not removed from the system; hence the persistent acidic pH as the setting reaction proceeds.

MgF_2_ (Equation (3)) will ionise to Mg^2+^ ions, which will contribute to the cross-linking process along with Ca^2+^ ions. Presumably, the simultaneous reactions of Equations (1)–(3) will determine the pH at any given time.

The effect of varying the MgO content on ExII is very apparent, confirming the role of the Mg(OH)_2_ formed. Previously reported results for a study including ExII and ExII F1 [21] showed that MgO also affected other physico-mechanical properties. Shore A hardness on setting for ExII was 31.6 compared to 21.1 for ExII F1. Tear energy (J/m^2^) was enhanced by including MgO; 403 for ExII and 321 for ExII F1 and superior to the commercial materials tested. Setting time (seconds) was also significantly reduced by including MgO (240 for ExII compared to 362 for ExII F1). These results combined with those in the current contribution (the pH results of the 7 different experimental materials, ranging in the diatomaceous earth and MgO_2_ concentration; Figure 1), confirm the dual role of MgO, an issue to be addressed when considering clinical applications. In summary MgO, (i) acts as a pH modifier by reacting with ‘F’ as HF is formed, thus increasing the pH of the alginate on setting; and (ii) Mg^2+^ additionally cross-links the alginate (the other cross-linking ion being Ca^2+^) [21].

### 2.4. Virucidal Assays

Experimental alginates with pH values ranging from 4.26 to 8.13 were challenged with HSV-1 and their antiviral capacity assessed by means of a virucidal assay. This involved incubating viral suspensions with cells from the Vero cell line and observing any cytopathic effect induced by exposure of the virus to the alginate materials.

For this study, a viral log reduction of 2.0 (−log_10_TCID_50_) was considered significant [22]. Experimental formulations ExII F1 and ExII F2 were able to reduce the viral titre of HSV-1 at all-time points, with the former performing slightly better overall (both achieved a viral log reduction of 2.0 (−log_10_TCID_50_ at the 5 min time point). This behaviour appeared to be time-dependent, as the antiviral activity of both materials increased with time. The average pH values of ExII F1 and ExII F2 before incubation with Vero cells were 4.28 and 4.39 respectively. HSV-1 requires a mildly acidic pH (~6.2) for release from the host cell membrane into the cell [23]. However, the antiviral activity of these materials suggests that low pH has an adverse effect on the virus. This has been discussed previously by [24], who demonstrated that entry of HSV-1 into Hep-2 cells was at least 100-fold less efficient at pH 6.3 than at pH 7.4.

It is postulated that the antiviral effect observed here, is the result of a pH-dependent conformational change induced in the virus upon exposure to the materials, inhibiting viral entry to the Vero cells. HSV-1 relies on the binding ability of glycoproteins found in its envelope (namely gC, gB and gD), to cellular receptors on the host cell [25]. Thus, it is suggested that conformational changes take place in either of these molecules, subsequently impeding host cell entry.

Materials ExII F3–F7 were able to reduce the viral load, but to a lesser extent than ExII F1 and ExII F2. ExII had reduced the viral load by 2.0 (−log_10_TCID_50_) at the 5 min time point and by 2.5 (−log_10_TCID_50_) at the remaining time points. The pH of this material before incubation with the Vero cells was 8.13, indicating that in this case, antiviral action was not low pH-mediated.

Hence, the acid pH of the experimental alginates induced a cytotoxic effect on HSV-1 on exposure, which was more effective with decreasing pH. The maximum log reduction achieved by the experimental alginates was 4.0.

## 3. Experimental Section

### 3.1. Materials

The commercial materials used were: Neocolloid, Zhermack and Empress, PSP Dental Company, Kent, UK), both formulated with sodium alginate. Compositions and sources of constituents for the experimental materials are listed in Table 1, where ExII contains 10% MgO and ExII F1 contains no MgO.

**Table 1:** Composition of experimental materials and source of constituents (ExII contains 10% MgO and ExII F1 contains no MgO).

Component	Supplier	ExII (%w/w)	ExII F1 (%w/w)
Manugel^®^DJX	ISP Alginates Ltd., Kent, UK	14.00	14.00
Crystacast plaster	CFS Partnership, Cornwall, UK	9.00	9.00
Potassium fluorotitanate	Rose Chemicals Ltd., London, UK	3.00	3.00
Tetrasodium pyrophosphate	Sigma-Aldrich Co. Ltd., Dorset, UK	0.84	0.84
Diatomaceous earth	Sigma-Aldrich Co. Ltd., Dorset, UK	63.16	73.16
Magnesium oxide	Sigma-Aldrich Co. Ltd., Dorset, UK	10.00	–

### 3.2. Methods

#### 3.2.1. Sample Preparation

Commercial materials were mixed according to the manufacturer’s instructions and experimental materials were prepared by combining 10 g of powder with 23 mL of water at 23 °C and mixing for 45 s.

#### 3.2.2. pH Measurements

A Gelplas flat tip electrode was used with an Orion 720A pH meter for pH measurements. The meter was calibrated using commercially available buffers (Mettler Toledo, Process Analytics, Leicester, UK), as stipulated by the manufacturer.

Each material was mixed and dispensed onto a glass slide. The electrode was placed upright on the slide and clamped into place, ensuring good contact between the material and the electrode surface. Readings were taken immediately after mixing, every 30 s for 30 min and then every minute for 30 min (60 min in total). After this, there was little variation in pH. Three samples of each material were tested. All tests were carried out at 23 °C and the instrument was re-calibrated between analyses.

The formulation for experimental material ExII F1 (Table 2) was used to create a series of materials with a range of pH values, which were taken forward for the virology study. Seven different experimental materials, ranging in diatomaceous earth and MgO concentration, were prepared.

**Table 2:** Composition of experimental alginates with varying magnesium oxide (MgO) content for virology study (ExII contains 10% MgO and ExII F1 contains no MgO).

Component	Formulation						
	ExII F1	ExII F2	ExII F3	ExII F4	ExII F5	ExII F6	ExII F7
	%w/w						
Manugel^®^DJX	14.00	14.00	14.00	14.00	14.00	14.00	14.0
Crystacast plaster	9.00	9.00	9.00	9.00	9.00	9.00	9.00
Potassium fluorotitanate	3.00	3.00	3.00	3.00	3.00	3.00	3.00
Tetrasodium pyrophosphate	0.84	0.84	0.84	0.84	0.84	0.84	0.84
Diatomaceous earth	73.16	73.06	72.16	71.91	71.76	71.56	71.16
Magnesium oxide	0	0.10	1.00	1.25	1.40	1.60	2.00

#### 3.2.3. Assay to Determine Activity against HSV-1

The virucidal assays were performed by Retroscreen Virology Ltd. Neocolloid (London, UK) (9 g powder: 18 mL water), Empress (17 g: 35 mL) and experimental materials (10 g: 23 mL) were mixed, divided in six equal portions and transferred into six-well plates. A cone-bottomed centrifuge tube was placed into the material whilst it was setting, to create a cavity with a known surface area. All experiments were done in duplicate.

Once the materials had set, the HSV-1 suspension was added to each cavity and incubated at 23 °C for the following time periods: 5, 30, 60 and 240 min. A positive control was made by incubating HSV-1 with 1.5% dimethyl sulfoxide (DMSO), which has a known cytotoxic effect on HSV-1 [26]. The control compound was incubated with the virus for the same time periods as the alginates.

The reactions were terminated by adding a neutralising solution. Residual virus activity in the wells was then determined by titrating the test solutions against Vero cells. Test solutions were then incubated with Vero cells for five days at 35 °C.

Replication of HSV-1 on the Vero cells causes a cytopathic effect (CPE), characterised by observing ballooning or rounded cells. Following incubation, the plates were examined microscopically for CPE to determine the level of virus replication. The virus titre was subsequently calculated using the Kärber Method [27,28] and the results expressed as −log_10_TCID_50_ (tissue culture infective dose); the dose causing 50% of the cells to become infected.

#### 3.2.4. Statistical Analysis

An unpaired, two-tailed student’s t-test was used to analyse effect of MgO on pH in experimental materials. The Bonferroni correction method was used to account for the increased risk of committing a type I error that arises from making multiple comparisons. Results were deemed statistically significant if *p* < 0.05.

## 4. Conclusions

Preparation and testing of a number of experimental alginates formulated *ab initio* has demonstrated that pH modification through manipulation of magnesium ion concentration confers antiviral activity.

The antiviral effects of the experimental materials have been achieved without the addition of therapeutic agents or currently employed chemical disinfection processes. Therefore, the materials are able to self-disinfect against HSV-1. This is an important demonstration of the principle we set out to test and opens the way to further research to enhance the self-disinfecting potential of alginates and other dental materials, thus eliminating the need for chemical disinfection procedures.
